# Efferent Modulation of Stimulus Frequency Otoacoustic Emission Fine Structure

**DOI:** 10.3389/fnsys.2015.00168

**Published:** 2015-12-10

**Authors:** Wei Zhao, James B. Dewey, Sriram Boothalingam, Sumitrajit Dhar

**Affiliations:** ^1^L.E.K. Consulting, BostonMA, USA; ^2^Roxelyn and Richard Pepper Department of Communication Sciences and Disorders, Northwestern University, EvanstonIL, USA; ^3^Knowles Hearing Center, Northwestern University, EvanstonIL, USA

**Keywords:** otoacoustic emissions, stimulus frequency otoacoustic emissions, fine structure, auditory efferents, medial olivocochlear bundle

## Abstract

Otoacoustic emissions, sounds generated in the inner ear, have become a convenient non-invasive tool to examine the efferent modulation of cochlear mechanics. Activation of the medial olivocochlear (MOC) efferents has been shown to alter the magnitude of these emissions. When the effects of efferent activation on the detailed spectral structures of these emissions have been examined, a shift of the spectral patterns toward higher frequencies has been reported for distortion product and spontaneous otoacoustic emissions. Stimulus frequency otoacoustic emissions (SFOAEs) have been proposed as the preferred emission type in the study of efferent modulation due to the simplicity of their production leading to the possibility of clearer interpretation of results. The effects of efferent activation on the complex spectral patterns of SFOAEs have not been examined to the best of our knowledge. We have examined the effects of activating the MOC efferents using broadband noise in normal-hearing humans. The detailed spectral structure of SFOAEs, known as fine structure, was recorded with and without contralateral acoustic stimulation. Results indicate that SFOAEs are reduced in magnitude and their fine structure pushed to higher frequencies by contralateral acoustic stimulation. These changes are similar to those observed in distortion product or spontaneous otoacoustic emissions and behavioral hearing thresholds. Taken together with observations made about magnitude and phase changes in otoacoustic emissions and hearing thresholds upon contralateral acoustic stimulation, all changes in otoacoustic emission and hearing threshold fine structure appear to be driven by a common set of mechanisms. Specifically, frequency shifts in fine structure patterns appear to be linked to changes in SFOAE phase due to contralateral acoustic stimulation.

## Introduction

Stimulus frequency otoacoustic emissions (SFOAEs) are low-level signals evoked by tonal probes, generated in the cochlea and recorded in the ear canal (Kemp and Chum, 1980). At moderate to high probe levels, SFOAEs arise from both linear coherent reflection and non-linear distortion mechanisms, characterized by long and short group delays, respectively (Shera and Guinan, 1999; Talmadge et al., 2000; Goodman et al., 2003). At low probe levels, linear coherent reflection is thought to dominate SFOAE generation (Zweig and Shera, 1995) with non-linear mechanisms theorized to be contributing at moderate and high probe levels (Talmadge et al., 2000). A quasi-periodic pattern, demonstrated in SFOAE level spectra, expected in phase and delay as well, is referred to as fine structure (Talmadge et al., 2000) or microstructure (Goodman et al., 2003). Multiple internal reflections in the cochlea, variation in effective reflectance along the cochlear partition, and the interaction between linear coherent reflection and non-linear distortion mechanisms have been implicated in generating and influencing SFOAE fine structure (Zweig and Shera, 1995; Talmadge et al., 2000). In guinea pigs, variation in effective reflectance along the cochlear partition accounts for the origin of SFOAE fine structure with moderate probe levels, whereas interference between SFOAE components of long and short phase-gradient delays account for SFOAE fine structure at higher probe levels (Goodman et al., 2003). Multiple internal reflections generate the fine structure in both the amplitude and phase of the basilar membrane transfer function in sensitive chinchilla cochlea (Shera and Cooper, 2013). In humans, knowledge of the origin of SFOAE fine structure as well as its probe level-dependency has potential clinical implications and can lead to the selection of optimal test conditions for the assessment of the cochlea as well as the auditory efferents.

Activation of the medial olivocochlear (MOC) efferents reduces the gain of the cochlear amplifier thereby decreasing the input to the auditory nerve (Galambos, 1956; Fex, 1962; Murugasu and Russell, 1996; Cooper and Guinan, 2003). MOC efferents have traditionally been associated with many possible functional roles, such as protection against acoustic trauma, facilitation of speech perception in noise, and auditory attention (for review, see Guinan, 2006). More recently, MOC efferents have been demonstrated to delay age-related changes in the cochlea (Liberman et al., 2014), play a role in perceptual learning (de Boer and Thornton, 2008), and be associated with localization in the presence of background noise (Andeol et al., 2011; Irving et al., 2011). SFOAEs have been employed for assessing the strength of MOC efferents (Backus and Guinan, 2007). However, the efferent influence on SFOAE fine structure has not been explored. Activation of the MOC pathway by contralateral noise not only alters the levels of OAEs, but also shifts distortion product otoacoustic emission (DPOAE) fine structure (e.g., Deeter et al., 2009), spontaneous otoacoustic emissions (SOAEs; e.g., Zhao and Dhar, 2010; Zhao and Dhar, 2011), and even hearing threshold fine structure (e.g., Dewey et al., 2014) toward higher frequencies. Since the fine structures of OAEs and hearing thresholds as well as the spacing between SOAEs are thought to originate from a set of common mechanisms involving mechanical resonance and multiple internal reflections in the cochlea, the MOC efferents should be expected to alter SFOAE fine structure in similar ways.

In this study, we recorded SFOAEs in humans at low to moderate probe levels (20 and 40 dB SPL) with and without activating the MOC efferents by a 60 dB SPL contralateral broadband noise. The origin of, and MOC influence on, SFOAE fine structure were explored.

## Materials and Methods

### Subjects

Eleven subjects (9 female and 2 male) between 21 and 31 years of age with normal hearing thresholds in both ears (20 dB HL or better at octave frequencies between 250 and 8000 Hz) participated in the experiment. These eleven subjects were chosen from a pool of over 50 specifically because their middle ear acoustic reflex thresholds, measured using broadband noise in the contralateral ear using a clinical impedance audiometer, were higher than 90 dB SPL. SFOAEs were recorded in one ear per subject. All procedures were approved by the Northwestern University Institutional Review Board. A written, informed consent was obtained from each subject. Measurements were conducted in a sound-treated audiological test booth.

### Signal Generation and Recording

Stimuli were generated by a Macintosh computer connected to a MOTU 828 MKII I/O device (sampling rate 44100 Hz, 24 bit), amplified, and presented via transducers (MB Quart 13.01 HX) coupled to an Etymotic Research ER-10B probe assembly using custom software. Signals from subjects’ ear canals were passed from the ER-10B microphone to a preamplifier (20 dB gain), then digitized by the MOTU and stored on disk for analysis.

### Measurement Procedure

Stimulus frequency otoacoustic emissions were obtained via the compression method (Kemp and Chum, 1980) using tones swept in frequency. A probe tone (20 and 40 dB SPL) and a compressor tone (60 dB SPL) were swept from 800 to 1800 Hz at a rate of 20 s/octave. MOC activity was elicited by a 60 dB SPL contralateral broadband noise (100–10000 Hz). A total of five conditions were interleaved: probe alone at 20 or 40 dB SPL, compressor alone at 60 dB SPL, probe at 20 or 40 dB SPL paired with contralateral noise. Six runs per condition were recorded and averaged. The total ear canal pressure at the probe frequency was estimated using a custom least-squares fit algorithm (Long and Talmadge, 1997).

### Analysis

**FIGURE 1:**
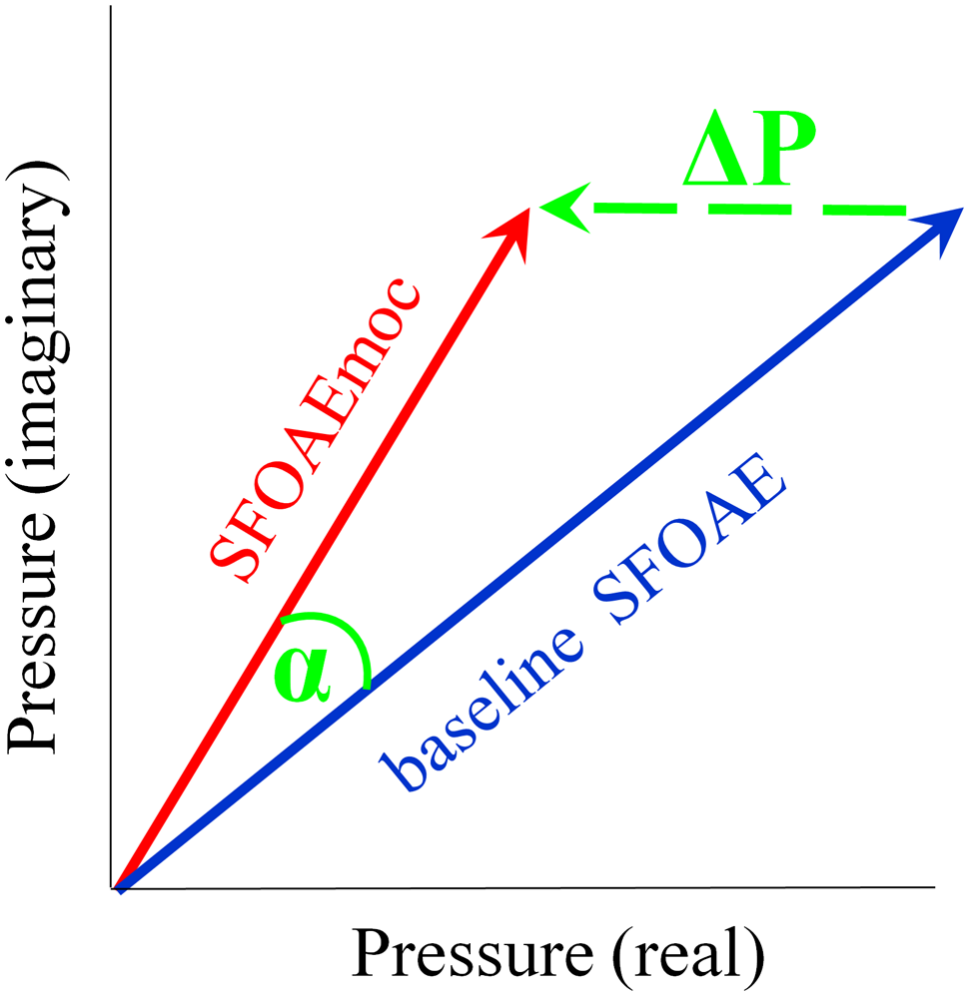
**Schematic of medial olivocochlear (MOC) modulation of stimulus frequency otoacoustic emissions (SFOAE).** SFOAE without and with contralateral noise are denoted as baseline SFOAE (blue) and SFOAEmoc (red), respectively. MOC effects were assessed via both the vector pressure change ΔP (green) and phase change α.

Stimulus frequency otoacoustic emissions were extracted by scaling the ear canal complex pressure in the compressor condition and subtracting it from that recorded in each of the other conditions. SFOAE without and with contralateral noise are denoted as baseline SFOAE and SFOAEmoc, respectively (**Figure 1**). The magnitude and phase of the vector pressure change between the two are denoted as ΔP and α. Throughout the paper, baseline SFOAE, SFOAEmoc and ΔP are color coded using blue, red and green traces, respectively.

**FIGURE 2:**
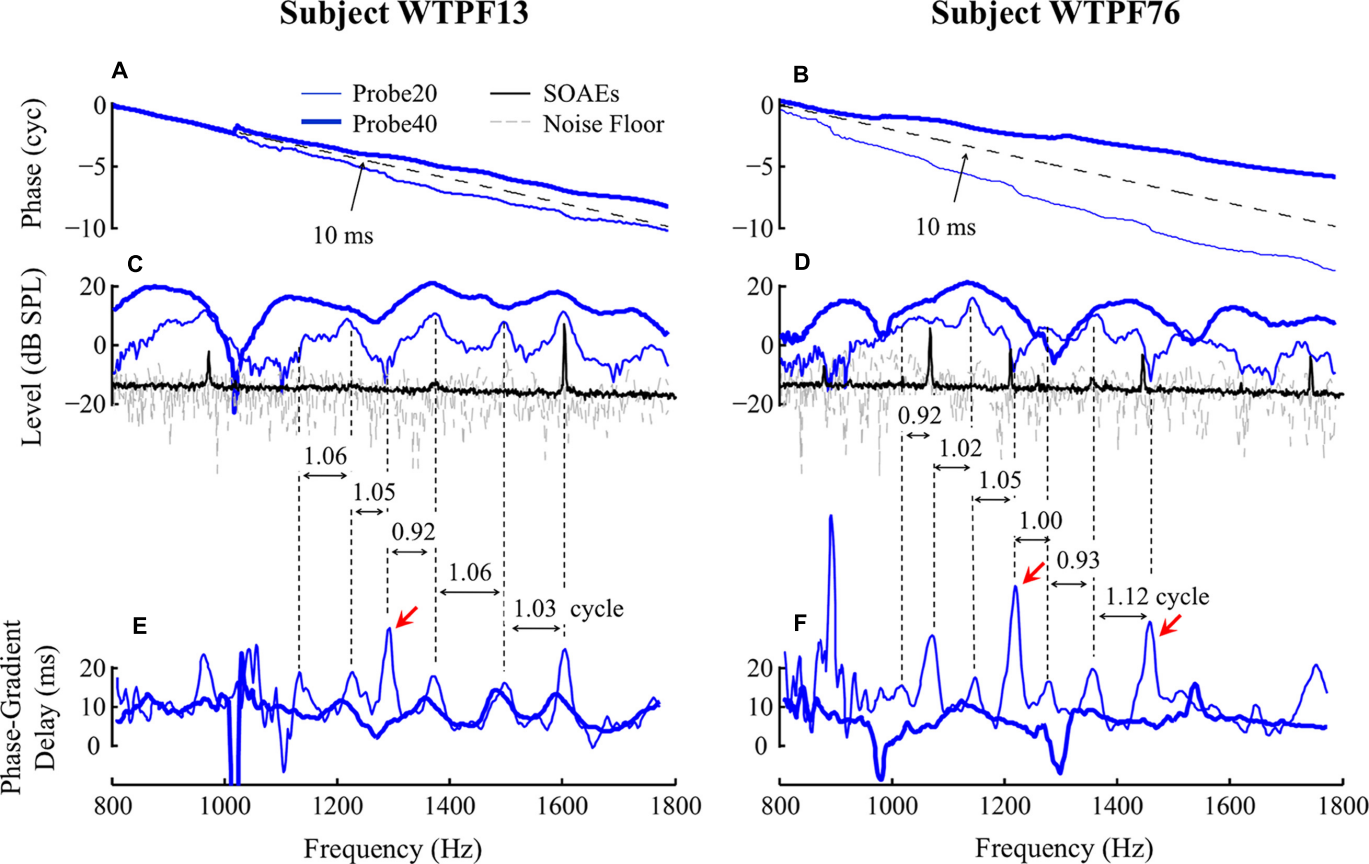
**Baseline SFOAE as a function of probe frequency for subjects WTPF13 and WTPF76 (columns).** Data obtained using 20 and 40 dB SPL probes are represented using thin and thick traces, respectively. SFOAE phase slopes are parallel with the phase slope of 10-ms delay (dashed reference line in **A,B**). Fine structure patterns of SFOAE level **(C,D)** and phase-gradient delay **(E,F)** are presented along with SOAE in panels **(C)** and **(D)** using the black traces. Vertical dashed lines are used to mark the alignment between fine structure patterns of level and delay. Red arrows indicate (the less common) alignment between SFOAE level valleys and phase-gradient delay peaks.

Absence of middle-ear muscle (MEM) contraction was confirmed by a phase-gradient delay of ΔP around 10 ms near 1500 Hz (see **Figures 2A,B**) indicating that ΔP was dominated by MOC-mediated changes in SFOAE pressure, and not MEM-induced changes in the stimulus pressure reflected at the eardrum (Guinan et al., 2003). This elegant method of differentiating between MOC- and MEM-mediated changes takes advantage of the expected phase-frequency relationship of SFOAEs versus middle ear reflectance. The phase gradient delay of the change vector is expected to be around 10 ms only when the change in ear canal pressure is actually due to a change in the SFOAE. In contrast, the phase gradient delay is expected to approximately 0 ms when the change in ear canal pressure is due to a change in middle ear reflectance caused by an MEM reflex.

**FIGURE 3:**
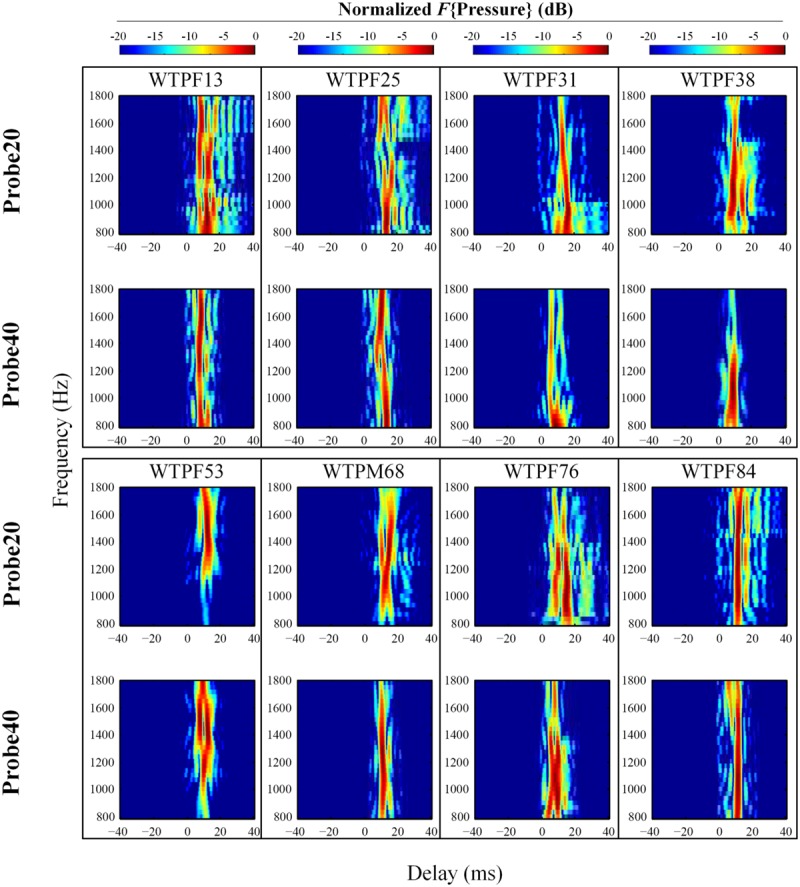
**Normalized results of inverse Fast Fourier Transform (IFFT) analysis on baseline SFOAE in eight subjects for 20 and 40 dB SPL probes.** In order to evaluate the weighting of components with different phase-gradient delays, each heat plot was normalized to its own maximal value. Only signals within 20 dB of the peak were retained. SFOAE components with different phase-gradient delays appear as vertical color bands on the SFOAE frequency versus phase-gradient delay heat plots.

**FIGURE 4:**
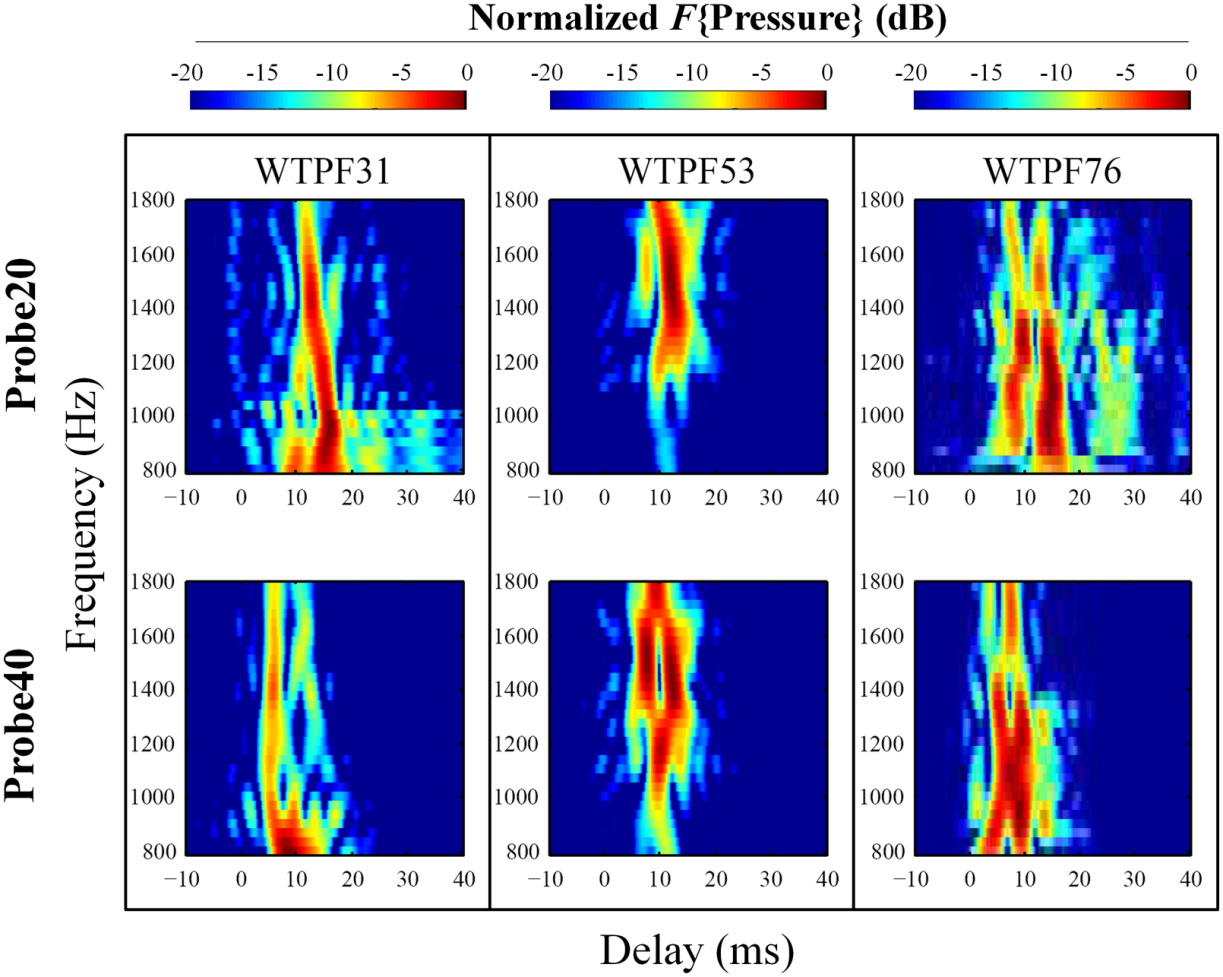
**Expanded heat plots of data from subjects WTPF31, WTPF53, and WTPF76 from **Figure 3**.** SFOAE components with delays less than 10 ms are almost exclusive to the 40 dB SPL probe (bottom row).

The extracted complex ear canal SFOAE estimate was converted from the spectral to the temporal domain by performing an inverse Fast Fourier Transform (IFFT, 300-Hz Hann window, 20-Hz steps; Kalluri and Shera, 2001). The magnitude of the IFFT output was normalized to its own maximal value in order to assess the weight of SFOAE components with varying delays, which appear as a vertical bands of energy separated in time (see **Figure 3**). The output of the IFFT analysis was found to be sensitive to parameters such as the width of the analysis window and the degree of overlap between adjacent windows. These parameters significantly affected the number of vertical bands of energy observed and the gap in delay between them. We developed confidence in the results of the IFFT by subjecting synthetic data with known reflections and delays to the analysis. To be further conservative in our approach, we limited the included data to those within 20 dB of the peak in all cases. Even after adopting this cautious approach we recommend that the reader attach limited value to the specific number of vertical bands of energy and the delays between them in **Figures 3, 4, and 6**.

### Equivalence of SFOAE Extraction Methods

To assess our method of using probes swept in frequency, we compared SFOAEs extracted by using discrete-frequency vs. swept-frequency tones, and by the compression versus suppression methods in two subjects. For discrete tones, the following triplet was applied, each portion of the triplet lasting 2 s: a 20/40 dB SPL probe tone, followed by a 20/40 dB SPL probe tone plus a 60 dB SPL suppressor 47 Hz below the probe frequency, followed by a 60 dB SPL compressor tone. This triplet was applied for probe frequencies between 800 and 1800 Hz in 20-Hz steps and repeated six times for each probe frequency. SFOAE recording and extraction via the compression method using swept-frequency tones was identical to the process described above. Discrete- tones and swept-frequency tones were interleaved to minimize probe drift across time. SFOAEs extracted by the suppression method using discrete-frequency tones, by the compression method using discrete tones, and by the compression method using swept-frequency tones were indistinguishable (**Supplementary Figure S1**) as has been demonstrated before (Kalluri and Shera, 2007b, 2013). It should be noted that the equivalence between otoacoustic emissions recorded using swept- and discrete-frequency tones is heavily dependent on various signal characteristics and analysis variables. The rate of frequency sweep is one such important variable and our claim of the equivalence of results between swept- and discrete-frequency tones is made specifically and only for slow frequency sweeps (20 s/octave) used here. In contrast, AlMakadma et al. (2015) have recently demonstrated differences in DPOAEs recorded using fast (1 s/octave) sweeps in stimulus frequency depending on the direction of the frequency change.

## Results

### Baseline SFOAE Fine Structure: Manifestations in the Spectral and Temporal Domains

Examples of baseline SFOAEs evoked by two probe levels (20 and 40 dB SPL) between 800 and 1800 Hz from two subjects are presented as a function of probe frequency in **Figure 2** for subjects WTPF13 and WTPF76. Thin and thick lines represent SFOAEs evoked by 20 and 40 dB SPL probes, respectively. For both probe levels, the SFOAE phase slope was approximately parallel with the reference line of a 10 ms delay (**Figures 2A,B**), indicating the dominance of a generation mechanism consistent with the properties of coherent reflection (Shera and Guinan, 1999). SFOAE phase slopes from all eleven individual subjects included in this study are shown in **Supplementary Figure S2**. Each of these exhibit a phase slope of approximately 10 ms with occasional discontinuities at unpredictable frequencies. On informal inspection, SFOAE phase slopes for the 20 dB SPL probe seem steeper than those with the 40 dB SPL probe.

Stimulus frequency otoacoustic emissions levels presented in **Figures 2C,D** appear to display a quasi-periodic fine structure. Not only does the 40 dB SPL probe yield higher level SFOAEs, the two probes also yield SFOAE fine structure with different morphologies, in location of peaks, spacing between them, as well as peak-to-valley depth. For subject WTPF13, SFOAEs evoked by the 20 dB SPL probe demonstrate good alignment between local peaks in SFOAE level and SOAEs (black trace in **Figure 2C**). Phase accumulation between adjacent SFOAE level peaks is around one cycle (**Figure 2C**). Similar phase accumulation between adjacent SFOAE level peaks can also be observed for subject WTPF76 (**Figure 2D**). However, the alignment between SOAEs and SFOAE level peaks is less evident for this subject (**Figure 2D**). The relationship between the location of SFOAE level peaks, SOAEs, and the phase accumulation between them is not as clear for SFOAEs evoked by the 40 dB SPL probe.

Stimulus frequency otoacoustic emissions phase-gradient delay, computed as the negative of the phase slope, is presented as a function of probe frequency in **Figures 2E,F** for probe levels of 20 and 40 dB SPL using thin and thick traces, respectively. The SFOAE delay versus frequency function also demonstrates periodicity that resembles the SFOAE level-frequency fine structure. SFOAEs evoked using the 20 dB SPL probe are associated with more prominent fine structure in both level and delay than those with the 40 dB SPL probe. For the 20 dB SPL probe, local peaks of the SFOAE delay-frequency function were most commonly aligned with local peaks of the SFOAE level-frequency function. However, delay peaks were occasionally also found to be aligned with level valleys (marked by red arrows in **Figures 2E,F**). Phase accumulation between adjacent peaks of the SFOAE delay-frequency function was approximately one cycle as demonstrated by the vertical dashed lines.

The temporal-domain representation of SFOAE pressure yielded by inverse Fourier analysis is represented in **Figure 3** for eight subjects, for 20 and 40 dB SPL probes. *F*{Pressure} is presented in dB and color-coded. Each vertical color band corresponds to an SFOAE component with a distinct delay. Each raw heat plot resulting from the Fourier transform is normalized to its own maximal value for better assessment of the relative strength of SFOAE components of different delays. Only data within 20 dB of the peak were included. This resulted in the rejection of some artifacts of the Fourier analysis, which manifested as ripples in the temporal domain.

Stimulus frequency otoacoustic emissions recorded using a 20 dB SPL probe receive greater contribution from SFOAE components with longer delays (sometimes up to 40 ms), whereas components of SFOAEs recorded using a 40 dB SPL probe cluster around 10 ms (**Figure 3**). Multiple, evenly spaced, bands can be observed for SFOAEs recorded using the 20 dB SPL probe. In some subjects, components with delays around 5 ms are demonstrated only in SFOAEs evoked by the 40 dB SPL probe (**Figure 4**).

### Efferent Influence on SFOAE Fine Structure

**FIGURE 5:**
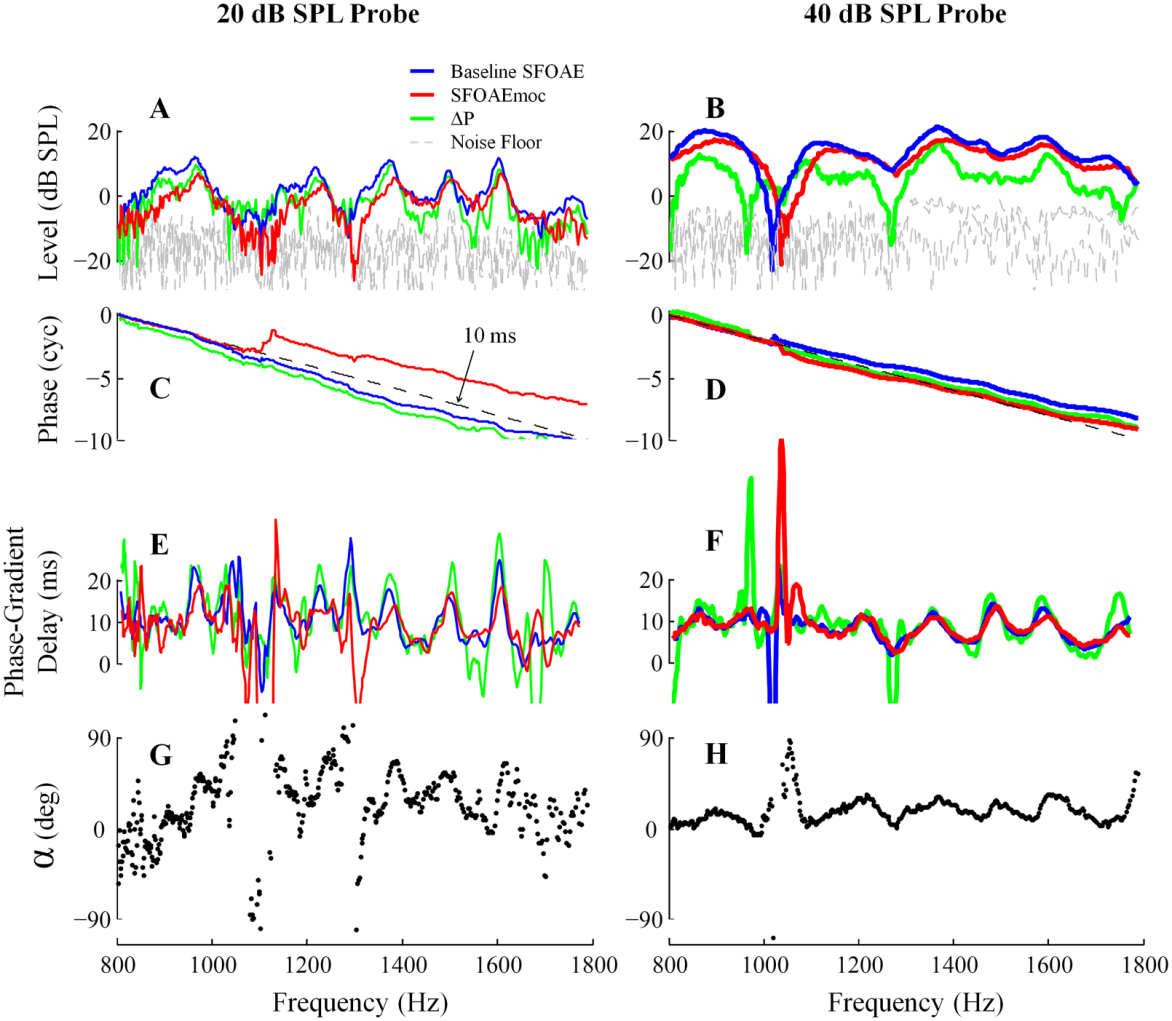
**Medial olivocochlearmodulation of SFOAE as a function of probe frequency for subject WTPF13.** Level, phase, delay, and phase change (α) are presented in separate rows with data from 20 and 40 dB SPL probes in the two columns. Representations of the baseline SFOAE, SFOAEmoc, and ΔP are made using different colors.

Efferent influence on SFOAE responses for both 20 and 40 dB SPL probes from subject WTPF13 is presented in **Figure 5**. Baseline SFOAE, SFOAEmoc, and ΔP are presented in blue, red, and green traces, respectively. MOC activity was elicited by a 60 dB SPL contralateral broadband noise. Absence of MEM contraction was confirmed by a phase-gradient delay of ΔP near 10 ms (**Figures 5C–F**). MOC activation suppresses SFOAE levels and shifts SFOAE fine structure laterally toward higher frequencies (**Figures 5A,B**). The phase slope of baseline SFOAE, SFOAEmoc and ΔP are parallel with the dashed reference lines representing a delay of 10-ms (**Figures 5C,D**). The phase-gradient delay versus frequency function is also shifted toward higher frequencies by MOC activation (**Figures 5E,F**). Finally, the SFOAE phase change α also exhibits periodicity as a function of probe frequency (**Figures 5G,H**), much like the periodicity in the SFOAE level-frequency function (**Figures 5A,B**) and the delay-frequency function (**Figures 5E,F**). The phase change (α) is almost always a phase advance, reaching up to 90 degrees (**Figures 5G,H**).

**FIGURE 6:**
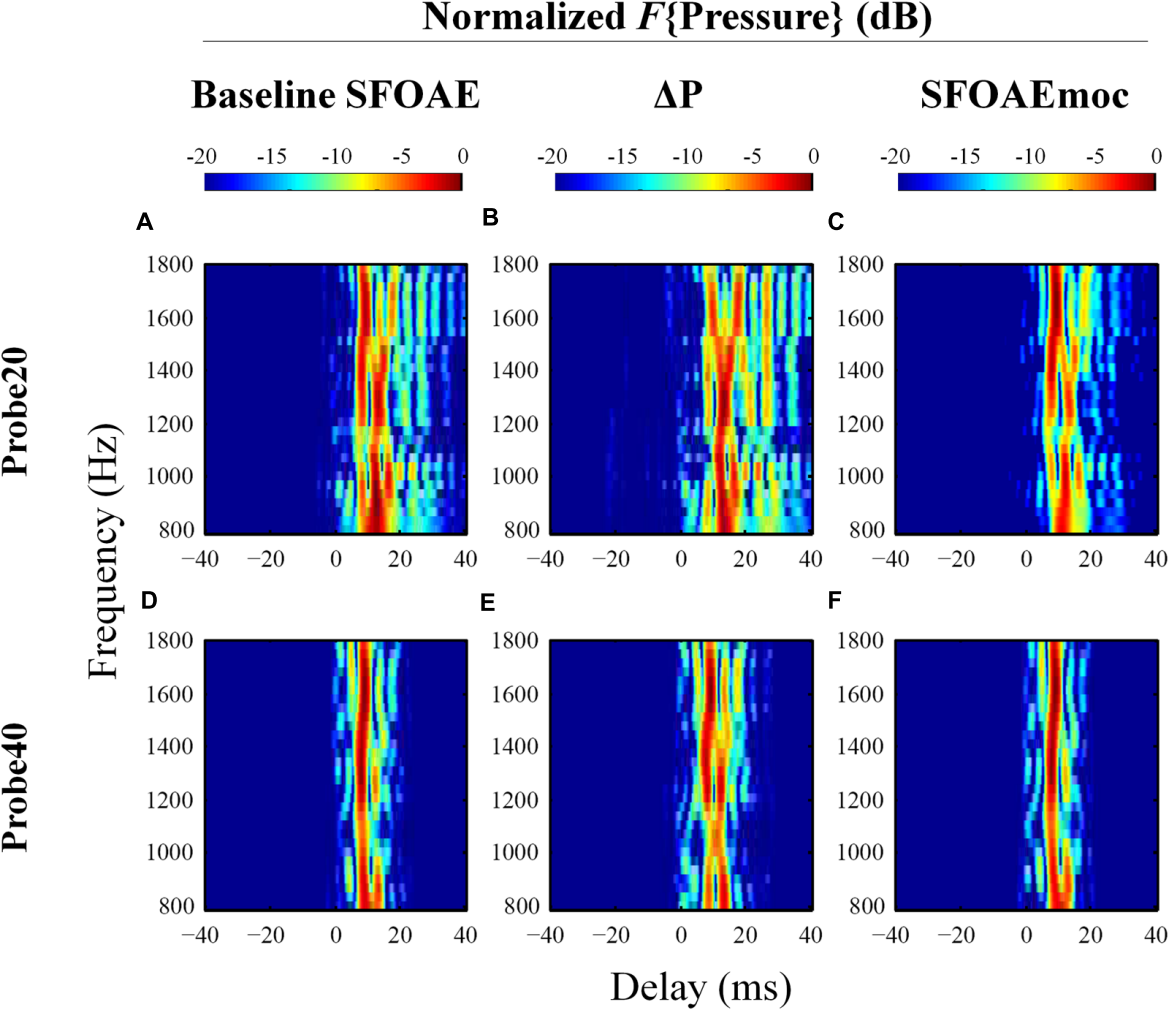
**Normalized results from IFFT analysis on baseline SFOAE (left column), ΔP (middle column) and SFOAEmoc (right column) from subject WTPF13.** Compared with baseline SFOAE, ΔP appears to receive greater contribution from components of delays above 10 ms.

Examples of the results of IFFT analysis on baseline SFOAE, ΔP and SFOAEmoc are presented in **Figure 6** (subject WTPF13). Comparing the first and last columns of **Figure 6** reveals the greatest differences between SFOAEmoc (right column) and baseline SFOAE (left column) for components with long delays. This trend is more prominent for the 20 dB SPL probe level. As a result, ΔP (middle column) is dominated by long-latency components (>10 ms) perhaps indicating a greater dependence of later reflections on cochlear gain and consequently greater inhibition upon MOC activation.

**FIGURE 7:**
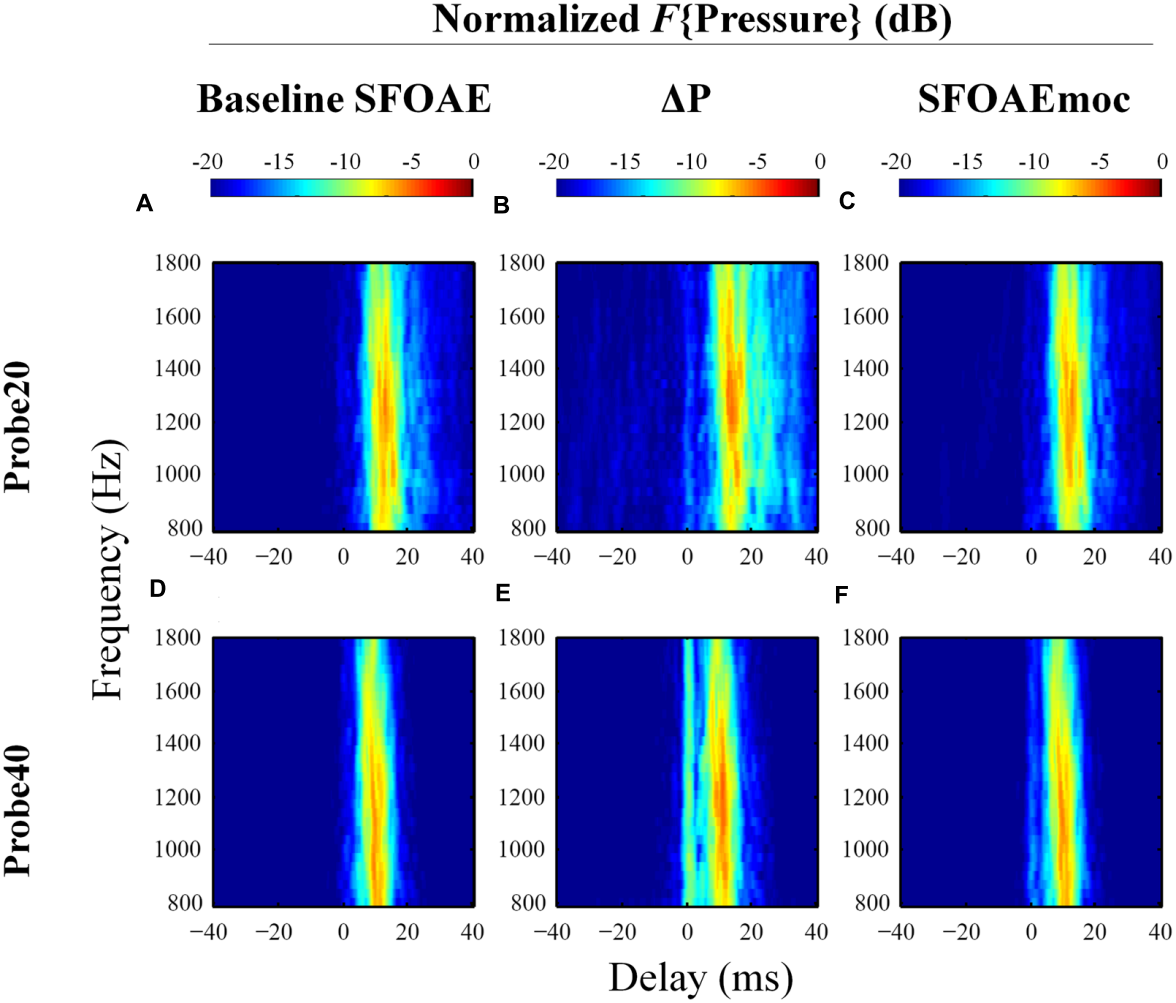
**Normalized IFFT results averaged across eleven subjects in a format similar to **Figure 6**.** Distinctive long-latency bands in individuals (**Figure 6**) are smeared after averaging across subjects. ΔP receives greater contribution from long-latency components than baseline SFOAE. A 0-ms band is observed in ΔP only for the 40 dB SPL probe.

Group results averaged across eleven subjects are displayed in **Figure 7**. Individual differences in the delays of discrete bands have a smearing effect on the average data, such that the dominant component near 10 ms in the average is wider than those from individual subjects (e.g., **Figure 6**), and no discrete bands are discernable above 20 ms. The contribution from components with delays greater than 10 ms is reduced for SFOAEs evoked by the 40 dB SPL probe as compared to those recorded using the 20 dB SPL probe. Interestingly ΔP exhibits a prominent band of energy near 0 ms in ΔP for the 40 dB SPL probe only (**Figure 7E**).

## Discussion

The goals of this study were to examine the modulation of SFOAE fine structure by the MOC efferent system. The general effect of MOC stimulation on SFOAEs has been studied extensively (e.g., Lilaonitkul and Guinan, 2009). SFOAEs and TEOAEs, both considered reflection emissions, have been preferred over DPOAEs in acknowledgment of the inherent complexity of DPOAE generation (multiple sites and multiple mechanisms; Guinan et al., 2003). SFOAEs are also available for study in a greater number of normal-hearing humans as compared to SOAEs. However, despite previous investigations into SFOAE fine structure, the components contributing to this fine structure, as well as their alteration by the MOC efferents have not been fully explored. Our results indicate that lower probe levels evoke contributions from SFOAE components with greater phase-gradient delays and these long-latency components are affected by efferent stimulation, more so than the shorter latency components. We also observed a shift of SFOAE fine structure toward higher frequencies, much like the observation in DPOAE fine structure (Abdala et al., 2009; Deeter et al., 2009), SOAEs (e.g., Zhao and Dhar, 2010), and behavioral threshold fine structure (Dewey et al., 2014).

### SFOAE Components

Processing the complex SFOAE pressure through an IFFT algorithm revealed roughly three groups of components segregated by differing delays (**Figures 3** and **4**). For both probe levels of 20 and 40 dB SPL, a prominent band of SFOAE energy was observed around 10 ms, consistent with previous observations (Kalluri and Shera, 2007a). Bands of SFOAE pressure at delays greater than 10 ms, often spaced regularly in time, were observed frequently for the 20 dB SPL probe condition (**Figure 3**). These can be interpreted as the outcome of multiple intracochlear reflections. Such reflections are predicted in various models of OAEs (Shera and Zweig, 1991; Dhar et al., 2002) and have been observed for DPOAEs and SFOAEs previously (Dhar et al., 2002; Goodman et al., 2003). Consistent with theoretical expectations, the band of SFOAE pressure around 10 ms was greater in magnitude as compared with the bands with greater delays. The dominance of a band of energy with a delay of approximately 10 ms in a frequency band around 1.5 kHz supports the notion of SFOAEs being generated near the peak of the traveling wave of the probe tone. This limited spatial distribution of SFOAE generators is consistent with model predictions (e.g., Shera and Guinan, 1999) as well as experimental results (Lichtenhan, 2012).

More prominent SFOAE components with delays shorter than 10 ms were observed for the 40 dB SPL probe condition. Often this early band of SFOAE energy was observed with a delay in the vicinity of 5 ms. This component with a shorter delay is consistent with the existence of sources of SFOAE basal to the peak of the traveling wave created by the probe (Mertes and Goodman, 2013; Charaziak and Siegel, 2015), the possibility of a fast mode of transport (as compared to a traditional mechanical traveling wave) of the SFOAE signal back to the ear canal (Siegel et al., 2005), as well as a non-linear generation mechanism (Talmadge et al., 2000). That the early latency component was most observable for the 40 dB SPL probe might suggest a greater contribution from a non-linear SFOAE generation mechanism, increased involvement of basal sources, or a combination of both at moderate probe levels.

### SFOAE Fine Structure

Fine structure was observed in both SFOAE level and group delay for both probe levels. The morphology of fine structure was different between the two probe conditions with narrower and perhaps better defined peaks and valleys observed more frequently for the 20 dB SPL probe condition (**Figure 2**). For this low probe level, the peaks in SFOAE group delay were separated by approximately one cycle of phase accumulation. These peaks in group delay were found to be aligned almost exclusively with peaks in SFOAE level and SOAEs. This observation would be consistent with the expectations from a model of global resonance leading to both SFOAE fine structure (at low probe levels) and SOAEs (Kemp, 1980; Shera, 2003). However, we occasionally observed peaks in SFOAE group delay for the 20 dB SPL probe condition to be aligned with valleys in SFOAE level (marked by red arrows in **Figure 2**). The origin of this association is unclear at this time.

For the 40 dB SPL probe condition, SFOAE fine structure appears to be more broadly spaced. The lack of multiple intracochlear reflections for this probe level along with a shorter delay for the main component suggests that the source of fine structure may be different for this probe condition as compared to that for a 20 dB SPL probe. In this case, SFOAE fine structure could arise from variation in the effective reflectance along the cochlear partition (Goodman et al., 2003) or due to interference between a linear reflection component and a non-linear distortion component (Talmadge et al., 2000; Goodman et al., 2003). Regardless, differences in SFOAE fine structure morphology with probe level may indicate level-dependent variations in the dominant SFOAE generation mechanisms and the relative contributions of multiple internal reflections.

### Efferent Modulation of SFOAE

Contralateral noise-induced MOC activity reduces SFOAE level (**Figures 5A,B**) and advances SFOAE phase (**Figures 5G,H**). The reduction in SFOAE level is consistent with a decrease in cochlear amplifier gain due to MOC activation. Long-latency components (>10 ms) that arguably correspond to multiple intracochlear reflections appear to be attenuated more than earlier components relative to the baseline by MOC activation (**Figures 6** and **7**). MOC activity reduces the SFOAE generating reflectance and the influence of this reduction is visible more in each successive reflection due to their roughly exponential dependence on the reflectance.

Medial olivocochlear-induced advance in SFOAE phase, denoted by α here, has previously been reported with coarse frequency resolution (Figure 5 in Francis and Guinan, 2010), and is consistent with the advance in basilar membrane vibration phase upon MOC activation (Murugasu and Russell, 1996). The quasi-periodic pattern observed when α is plotted as a function of frequency mimics that of the phase-gradient delay versus frequency function of the baseline SFOAE (**Figure 5**).

Medial olivocochlear activity shifts SFOAE fine structure toward higher frequencies (**Figures 5A,B**). Similar shifts have been observed in DPOAE fine structure (Abdala et al., 2009; Deeter et al., 2009) and SOAE frequency (Mott et al., 1989; Harrison and Burns, 1993; Zhao and Dhar, 2010). The models that account for DPOAE and SOAE shifts are based on a change in stiffness of the basilar membrane leading to a phase advance (Mott et al., 1989). Our results suggest a similar shift in SFOAE phase resulting in a shift in SFOAE fine structure toward higher frequencies. It can further be concluded that the shift in SFOAE phase is at the root of the shift toward higher frequencies of all evoked OAE fine structure, SOAEs, and even behavioral hearing threshold fine structure.

**FIGURE 8:**
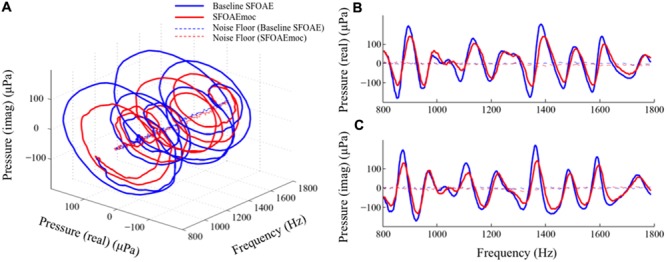
**Alternative views of MOC-induced SFOAE fine structure shift.** The complex SFOAE pressure is transformed into a spiral in three-dimensional space **(A)**. MOC modulation on this spiral can be dissected in two perpendicular directions: a radial shrinkage and an axial advance. Projecting this spiral to the pressure (real) – frequency plane **(B)** and the pressure (imaginary) – frequency plane **(C)** provides better views of MOC effects along the two perpendicular directions.

Plotting the two-dimensional SFOAE pressure vector as a function of probe frequency yields a spiral in a three-dimensional space (**Figure 8A**). The blue and red spirals are baseline SFOAE and SFOAEmoc, respectively. MOC modulation of SFOAE fine structure can be dissected in two perpendicular directions: shrinkage along the radial axis and shift along the axial axis. These changes are better visualized by projecting the two spirals to the real pressure-frequency plane and the imaginary pressure-frequency plane (**Figures 8B,C**). It is appealing to relate the shrinkage along the radial axis to the reduction in the cochlear amplifier gain, and the shift along the axial axis to the stiffness change of the basilar membrane that alters the cochlear characteristic frequency map.

A vertical band near 0 ms was found in the IFFT analysis of ΔP in some of the subjects (as in **Figure 6**) and in the averaged data (**Figure 7**). It would be convenient to associate this 0 ms component with activation of the MEM reflex. However, the phase gradient of ΔP did not indicate domination by the MEM reflex. Thus, even if the MEM reflex was activated in some subjects with the 40 dB SPL probe, it did not dominate the overall changes in SFOAE pressure. Alternately, it is also possible that MOC activation generated a non-linear distortion component, not documented before.

### Clinical Considerations

Disruption of, or changes in, the efferent modulation of otoacoustic emissions is of clinical interest. For example, efferent modulation of otoacoustic emissions is different in adults and children with learning disabilities (e.g., Garinis et al., 2008). The strength of efferent modulation is associated with the ability to locate a signal in space in the presence of background noise (Andeol et al., 2011; Irving et al., 2011; Liberman et al., 2014). Clinicians are expressing increasing interest in measuring efferent modulation of otoacoustic emissions for these reasons.

In typical clinical applications, TEOAEs or DPOAEs are measured with and without broadband noise in the contralateral ear and the difference in magnitude caused by the background noise is taken as a measure of the strength of efferent modulation of otoacoustic emissions. Shifts in DPOAE fine structure due to efferent stimulation causes significant complications in the interpretation of clinical data. Because the entire fine structure pattern is not evident in clinical measures at isolated frequencies, a shift in fine structure can manifest as an enhancement of DPOAE level due to efferent stimulation. If SFOAEs were used for clinical measures of efferent function, the same complications due to fine structure shift would be expected to complicate interpretation. In the absence of full characterization of fine structure, multiple measures at strategic frequencies near the frequency of interest could help avoid confusion, as at least one measure would then be expected to fall near the peak of fine structure yielding a stable estimate of efferent strength (see Deeter et al., 2009 for details). While we have focused on frequency shifts in fine structure as the source of the occasional enhancement in otoacoustic emission levels observed in the literature, another possible cause leading to the same effect in at least quadratic DPOAE levels (e.g., f_2_–f_1_) could be a change in the operating point of outer hair cells (Abel et al., 2009).

### Closing Summary

To the best of our knowledge, these results are the first demonstration of an MOC-induced shift of SFOAE fine structure. This observation not only accounts for occasionally observed SFOAE enhancement by contralateral noise, but also bears clinical relevance as to the selection of SFOAE probe frequency (peak vs. valley) for examining the strength of MOC efferents. Finally, we argue that the shifts in all OAE and behavioral fine structures are driven by a common source – efferent-induced changes in SFOAE phase.

## Conflict of Interest Statement

The authors declare that the research was conducted in the absence of any commercial or financial relationships that could be construed as a potential conflict of interest.

## Supplementary Material

Figure S1 **Comparison of stimulus frequency otoacoustic emissions (SFOAEs) obtained through the suppression and the compression methods using discrete or swept-frequency tones (Subjects WTPM18 and WTPF76).** SFOAE level yielded by the suppression method using discrete tones, the compression method using discrete tones, and the compression method using swept-frequency tones are represented by red, blue, and green colors, respectively.

Figure S2 **Stimulus frequency otoacoustic emissions phase slopes are distinctive across subjects (*N* = 11).** For both probe levels, phase slopes are approximately parallel with the 10-ms phase slope (dashed line).
