# Supplementary figures and images for: Efferent Modulation of Stimulus Frequency Otoacoustic Emission Fine Structure
Source: Front Syst Neurosci. 2015 Dec 10;9:168. doi: 10.3389/fnsys.2015.00168 (PMC4674573; doi:10.3389/fnsys.2015.00168)

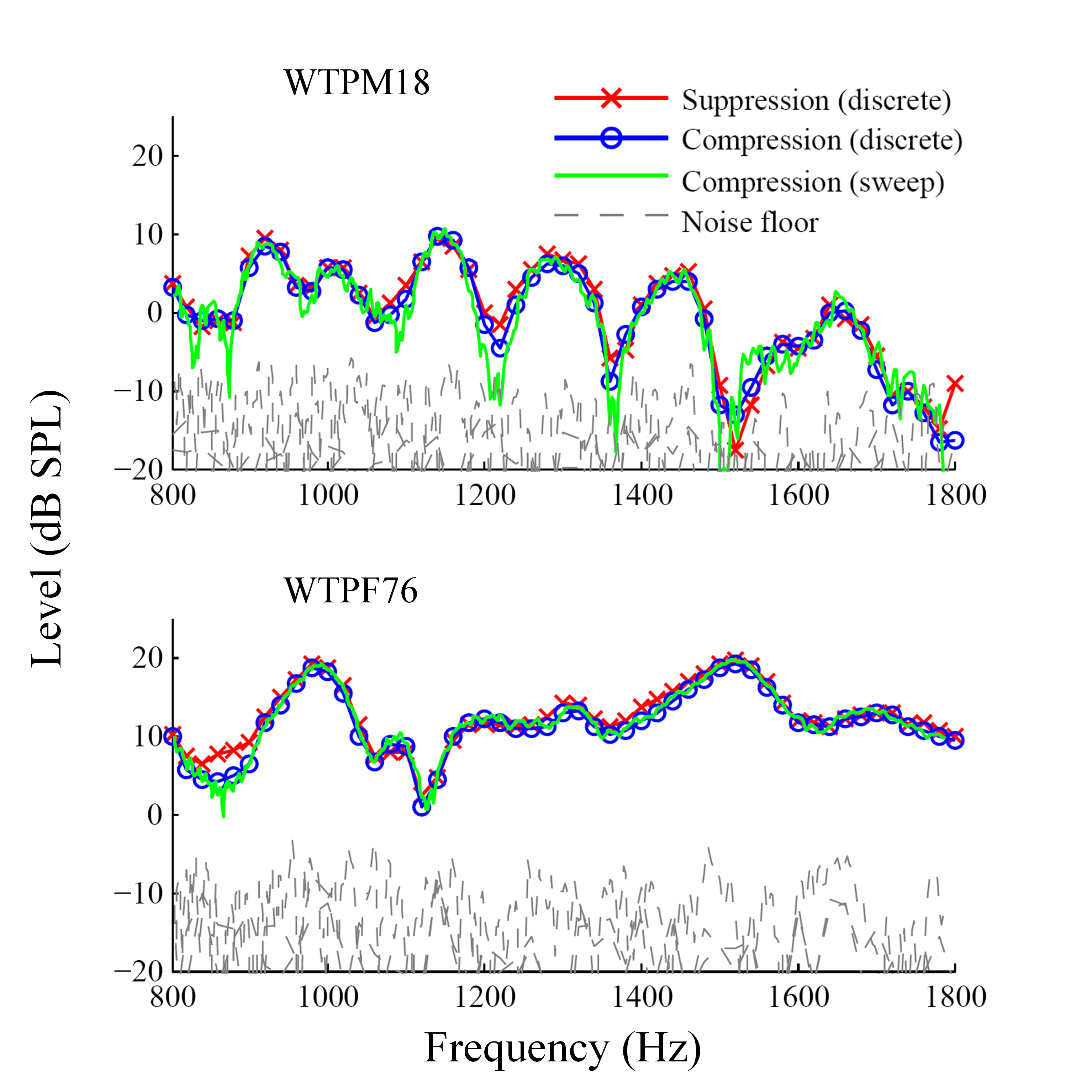

Supplement: Figure S1 — Comparison of stimulus frequency otoacoustic emissions (SFOAEs) obtained through the suppression and the compression methods using discrete or swept-frequency tones (Subjects WTPM18 and WTPF76). SFOAE level yielded by the suppression method using discrete tones, the compression method using discrete tones, and the compression method using swept-frequency tones are represented by red, blue, and green colors, respectively. [file Image_1.TIF]

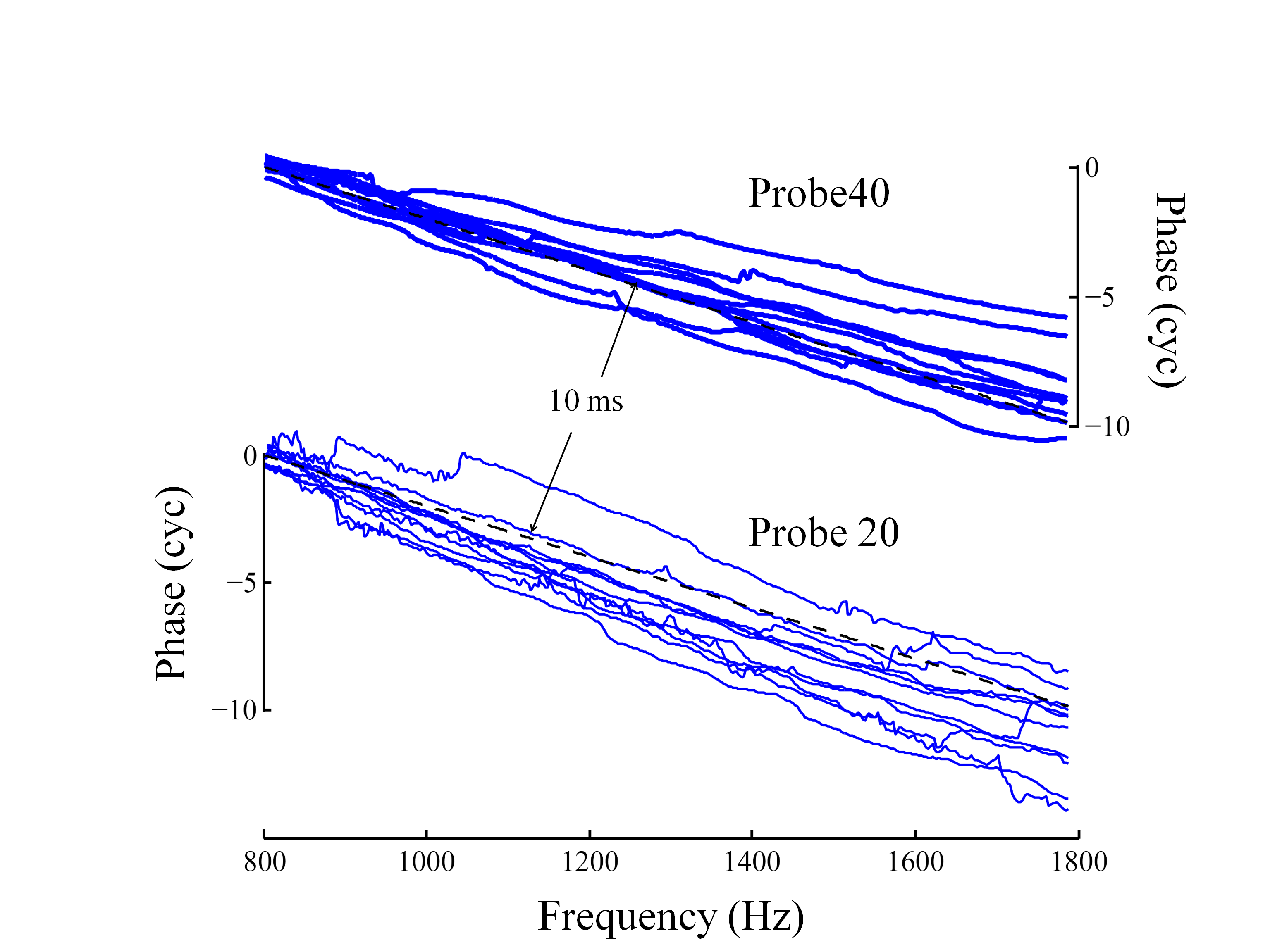

Supplement: Figure S2 — Stimulus frequency otoacoustic emissions phase slopes are distinctive across subjects (N = 11). For both probe levels, phase slopes are approximately parallel with the 10-ms phase slope (dashed line). [file Image_2.TIF]
